# 

**Published:** 1882-08

**Authors:** 


					﻿COLLABORATORS:
ALF. L. LOOMIS, M.D......... New	York.
FESSENDEN N. OTIS, M.$>.....
F. H. BOSWORTH, M.D.........
J. L. LITTLE, M.D...........
C. E. FRANCIS. D.D.S., M.D.S....
A. J. C. SKENE, M.D....Brooklyn,	N. Y.
C. A. MARVIN. D.D.S......
W. C. BARRETT. M.D.. D.D.S., Buffalo, “
EDWIN T. DARBY, M.D., D.D.S... .Phil., Pa.
P. S. WALES, M.D., Surg.-Gen’l U. S. Navy.
SAMUEL C. BUSEY, M.D.. Washington, D.C.
H. F. CAMPBELL, M.D......Augusta,	Ga.
C. H. MASTIN, M.D., LL.D...Mobile, Ala.
J. J. CHISOLM, M.D..... ..... Baltimore, McL
C. F. BEVAN, M.D........ “
I.	E. ATKINSON, M.D.....
H. McGUIRE, M.D.........Richmond, Va.
F. P. PORCHER, M.D.....Charleston, S. C.
J.	W. UNDERHILL, M.D.....Cincinnati, O.
JAS. T. WHITTAKER, M.D....
J. RANSOHOFF, M.D., F.R.C.S.
F. FORCHHEIMER, M.D......
A. R. JACKSON, M.D.......Chicago,	Ill.
S. V. CLEVENGER, M.D......... “	“
E. F. INGALS, M.D..........
J. H. CARSTENS, M.D....Detroit,	Mich.
PROSPECTUS, 1882.
The Independent Practitioner is cos-
mopolitan in its scientific researches in
Medicine, Surgery, Obstetrics, Dentistry,
Hygiene, and Popular Science, and gives
the refined findings to its readers. It is '
independent of colleges, cliques, isms, and
any advertising firm, and fearlessly renders
justice to all. Politics and religious creeds
are excluded.
				

